# A Wii Bit of Fun: A Novel Platform to Deliver Effective Balance Training to Older Adults

**DOI:** 10.1089/g4h.2015.0006

**Published:** 2015-12-01

**Authors:** Caroline Whyatt, Niamh A. Merriman, William R. Young, Fiona N. Newell, Cathy Craig

**Affiliations:** ^1^ School of Psychology, Queen's University Belfast, Belfast, United Kingdom.; ^2^ School of Psychology and Institute of Neuroscience, Trinity College Dublin, Dublin, Ireland.; ^3^ The Institute of Environment, Health and Societies, Brunel University London, London, United Kingdom.

## Abstract

***Background:*** Falls and fall-related injuries are symptomatic of an aging population. This study aimed to design, develop, and deliver a novel method of balance training, using an interactive game-based system to promote engagement, with the inclusion of older adults at both high and low risk of experiencing a fall.

***Study Design:*** Eighty-two older adults (65 years of age and older) were recruited from sheltered accommodation and local activity groups. Forty volunteers were randomly selected and received 5 weeks of balance game training (5 males, 35 females; mean, 77.18 ± 6.59 years), whereas the remaining control participants recorded levels of physical activity (20 males, 22 females; mean, 76.62 ± 7.28 years). The effect of balance game training was measured on levels of functional balance and balance confidence in individuals with and without quantifiable balance impairments.

***Results:*** Balance game training had a significant effect on levels of functional balance and balance confidence (*P* < 0.05). This was further demonstrated in participants who were deemed at high risk of falls. The overall pattern of results suggests the training program is effective and suitable for individuals at all levels of ability and may therefore play a role in reducing the risk of falls.

***Conclusions:*** Commercial hardware can be modified to deliver engaging methods of effective balance assessment and training for the older population.

## Introduction

Maintaining balance is a complex process that requires the integration of convergent information from the visual, vestibular, and proprioceptive sensory systems to regulate the oscillation of the body's center of pressure (COP), around the center of mass.^1^ With aging, however, the central nervous system does not appear to integrate sensory information as efficiently.^2^ Combined with reduced muscle mass, and thus strength, this underlying disintegration leads to the gradual degeneration of the balance control system in the elderly.^3^ Indeed, observed age-related increases of COP sway,^4^ COP velocity,^5^ and reduced limits of stability^6^ indicate compromised levels of balance control. This deterioration in balance control can have detrimental effects on the lives of older adults, leading to an increased likelihood of falling and reported “fear” of falling.^6–8^

Falls are a leading public health concern with statistics reporting that 30 percent of adults 65 years of age and older, and 50 percent of those 80 years of age and older, experience a fall annually.^9,10^ In 2013, falls were estimated to cost the United Kingdom's National Health Service in excess of £2.3 billion every year,^10^ with the U.S. Centers for Disease Control and Prevention, estimating that direct medical costs related to falls approached $34 billion for 2013.^11^ With global improvements in health and social care, these figures are projected to increase because of an inevitable shift in population demographics, with an increase in the at-risk sample.^10,11^ As such, the need to understand the complex nature of falls, as well as to design effective falls prevention and/or rehabilitation systems, has never been stronger.

Research demonstrates the potential of exercise in preventing falls in older adults and is now a fundamental element in public guidelines for falls prevention.^11,12^ This growing, evidence-based research highlights the importance of patient-orientated, balance-targeted exercises,^13^ which draw focus to a sense of body position in space.^14^ Indeed, feed-forward control by the central nervous system requires an accurate internal representation of the individual's limits of stability.^15^ Physiotherapists/physical therapists can thus help individuals train their central nervous system to respond to different sensory challenges through balance control exercises. This promotes better balance, and thus fewer falls. Unfortunately, this personal training can be extremely costly and is often considered tedious and repetitive, leading to high levels of attrition.^16–18^

An alternative way of engaging older adults in balance control exercises is to use technology that can monitor COP displacement, such as balance platforms, and to use this in a context that is both interactive and fun.^19–21^ Such studies contend that computer-generated games, used in a rehabilitation context, can induce significant improvements in general movement. Indeed, games that allow participants to progress through increasing levels of difficulty can enhance levels of intrinsic motivation through the use of external factors that facilitate the monitoring of improvement, as well as the creation of a patient-oriented goal.^16^ Moreover, real-time visual and auditory feedback can facilitate awareness of one's own body position (closing the loop with feed-forward control from the central nervous system), increasing motivation.^20^ In sum, these results demonstrate the potential of custom-made interactive posturography systems to provide an effective method of functional balance training for older adults.^20–22^ However, the use of such high-end research-grade balance platforms often requires specialist administration and analysis and therefore limits the widespread applicability of such gaming therapy in clinical practice.

The Nintendo Wii™ technology (Nintendo, Kyoto, Japan) is one example of a *commercially* available interactive system that is capable of reliably monitoring COP. The arrival of this gaming system was revolutionary, as it was the first to directly use larger amplitudes of body movement as a game controller. The popularity of Nintendo Wii technology as a clinical exergaming tool has been well documented.^23–32^ Clinical studies that examine the efficacy of the Wii technology to promote stability and mobility among older adults repeatedly demonstrate the usability of the system as a balance training tool.^23–32^ However, upon closer examination, issues regarding sample size, rigor of clinical assessment, and limitations in the structured training are highlighted. Indeed, several studies^23,26^ failed to statistically assess levels of functional improvement following game training. Moreover, those that used statistical procedures often used a limited sample size, preventing the generalizability of such findings.^24,27,28,30^ As noted by Goble et al.,^31^ 40 percent of studies included five individuals or fewer in assessments of the Nintendo Wii as a balance rehabilitation and training tool.

Moreover, several studies enforced a structured routine for gameplay, with participants required to play preselected games in an ordered manner.^23,24,26–30,32^ These games largely consist of yoga exercises such as deep breathing, as well as balance exercises such as table tilt (requiring controlled shifts in COP across all axes), ski slalom (requiring shifts in COP in the mediolateral plane), and soccer heading (requiring timed shifts in the anterior–posterior plane). To date, only a single study has allowed participants the opportunity to freely self-select gameplay.^25^ This reliance on selected games that often do not map COP changes accurately in the game environment, along with clinicians having to impose a preset structure, highlights problems around adapting the commercially available Wii interface to the target population.

Despite the frequent use of the Nintendo Wii as a balance training tool, few games are adapted to the action capabilities of older adults, who often experience restricted or limited mobility. This in turn creates limitations in the usability and feasibility of integrating commercial technology into clinical practice. Indeed, previous clinical assessment with small *n* populations may fail to adequately represent the movement, as well as cognitive capabilities, of the larger target population.

Clinician reports have highlighted the inability to tailor training programs through the Wii interface.^33–35^ This inability to alter underlying game parameters so that they target specific balance difficulties limits the patient-oriented experience.^35^ Moreover, the singular format can leave games inaccessible to some participants, who may find the physical and/or cognitive challenges embedded within these preset games daunting or frustrating.^35^ This is particularly pertinent, as reduced physical and cognitive ability are known to be leading factors in risk of falls.^36^ Therefore, it is suggested that preset game formats may lead to disengagement, excluding participants at high risk of falls from such therapeutic interventions. As highlighted by a recent review article,^37^ such difficulties with accessibility may result in a preference for more traditional methods of training. Indeed, results of clinical exergaming using preset game formats are limited and inconsistent in such high-risk groups.^24,38^

This is further confounded by confusion in the definition and classification of those deemed high risk. For instance, results from Agmon et al.^24^ are initially promising, demonstrating significant balance improvements in high-risk, older adults (with detectable balance impairment) following structured gameplay. However, upon closer inspection, issues with the use of a liberal threshold for high-risk classification, as well as levels of improvement, are highlighted. In particular, Agmon et al.^24^ categorized and quantified balance impairment using the Berg Balance Scale (BBS),^39^ a standardized functional balance assessment tool, with a high-risk threshold of ≤52. Debate surrounds the use of such liberal thresholds, with a more stringent threshold of ≤45 encouraged.^40^ Moreover, an average improvement of 4 points on the BBS, although deemed statistically significant, is lower than the minimal detectable change threshold of 5 points.^39,41^ Such underlying issues, combined with the traditional use of a small *n* population (*n* = 7), raises questions over the usability of such balance gaming systems with older adults deemed at high risk of falls and demonstrating quantifiable balance impairments.

In addition to providing a method of balance training, the Wii Balance Board (WBB) hardware can effectively assess levels of postural control through kinematic variables such as COP displacement.^42,43^ As such, this technology may provide a low-cost, portable assessment device to complement traditional methods of balance assessment, such as the BBS,^40^ Timed Up and Go,^44^ and functional reach tests.^45,46^ Indeed, research-grade balance platforms for kinematic assessment are often immobile and highly expensive (e.g., NeuroCom^®^ Balance Master^®^ [Neurocom International Inc., Clackamas, OR] systems^47^), reducing their clinical applicability. Few studies have examined the reliability of the commercial Wii interface software such as the “Wii Fit Age” tests to quantify postural control, specifically changes in postural control.^31,48^ Despite the efficiency of the WBB, this evidence would indicate that commercial interfaces, such as the “Wii Fit Plus” balance tests, fail to reliably encapsulate underlying physiological changes in balance control, mobility, and related changes in balance confidence.^48^

In light of prior research, the aim of this project was to design, develop, and deliver a balance assessment and game training protocol that exploited the usability of WBB technology but that was sensitive to the movement capabilities of an older population. Specifically, this project aimed to provide a unique user interface through which a clinician can directly modify game parameters, simultaneously challenging participants while ensuring inclusion of all individuals, namely, those at low and high risk of falls.

## Materials and Methods

### Participants

Eighty-four older adults were recruited from sheltered accommodation and retirement groups in the local community. Forty participants (5 males, 35 females) completed the intervention (2 dropped out, both female) and made up the experimental group (mean age, 77.18 ± 6.59 years), whereas 42 participants (20 males, 22 females) kept activity diaries for the same period and made up the control group (mean age, 76.62 ± 7.28 years). Note there was a considerable presence of females within the experimental (training) group. This was coincidental and a by-product of random assignment to control and training groups. However, it must be noted that such a pattern should not negatively affect results. Indeed, as the impact of muscular power/strength is traditionally thought to affect balance, male dominance in these arenas should result in a stronger, more effective control group. As such, any significant gains made by the training group—particularly those in relation to the control group—may be viewed as all the more promising.

Inclusion criteria included the following: normal or corrected vision and hearing, no history of primary medical risk factors for falls, prescreening results of ≤54 for the BBS^40^ and ≥24 on the Mini Mental State Exam (MMSE),^49^ and age of ≥65 years. Note that prior to commencing training, all participants were provided with test stimuli to ensure that all features were easily visible and fully audible. Those with profound difficulties were excluded from participating.

### Balance training and assessment system

**FIG. 1.**
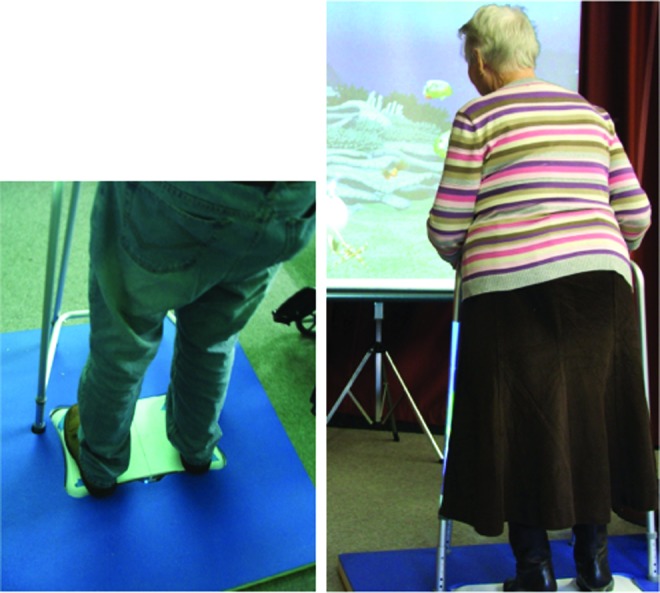
Apparatus used during the assessment of functional balance and the delivery of balance game training. A surround platform was included to provide additional safety. It should be noted that if participants used the safety surround during balance assessment, this was recorded and later controlled for. (Color graphics available at www.liebertonline.com/g4h)

The hardware for the balance training and assessment system comprised a commercially available WBB, a laptop, a surround foam platform with plywood base, and a Zimmer frame embedded in the foam platform (Fig. 1). This resulted in a safety platform, to minimize fall risk. The computer-generated visual displays that made up the system provided the visual impetus allowing users to control their balance and general movement. The screen was 1.09 m in height and 1.79 m in width with a throw of 2.7 m between the screen and the participant. All training occurred in a well-lit room. The WBB provided the means by which the user interacted with the assessment and training system.

### Development of a user interface

A custom-made interface (between the WBB and the laptop) functioned so that COP data streamed wirelessly via Bluetooth^®^ (Bluetooth SIG, Kirkland, WA) connectivity from each of the four pressure sensors under the WBB, allowing participants to use their balance as a controller. This made it possible to access its pressure sensor data through four 16-bit interfaces, along with calibration data, to allow for conversion to mass measurements. These data were not encrypted and therefore did not require a time-consuming encryption/decryption process. The personal computer program acted as a memory-map repository of the WBB data, allowing the three-dimensional graphics software Virtools to interface and poll the data dynamically at 40 Hz. 3DVia Virtools version 4.1 (Dassault Systèmes, Forest Hill, MD) software was chosen to develop the platform's game logic, whereas Autodesk (San Rafael, CA) 3D Studio Max 2011 was used to create the three-dimensional objects, and Adobe Photoshop CS 5 (2010; Adobe Systems, San Jose, CA) was used to create flat image resources such as menus, background images, and textures. Virtools displayed the interface via an OpenGL version 2.0-compatible graphics card and drivers, with stimuli displayed at 80 frames per second using a resolution of 1024 × 768 in 16-bit color.

Real-time data were polled from the WBB at a rate of 40 Hz using a custom-made algorithm, allowing for the constant updating of participant movement with no perceptible gap or lag. COP displacement in the mediolateral (*x*) and frontal planes (*y*) was calculated using the following equations:

\begin{align*}px \frac { ( TR \ \mid \ VR ) \ ( TL \ \mid \ BL )
}  { TR \ \mid \ BR \ \ \ \ TL \ \mid \ BL } \end{align*}

\begin{align*}py \frac { ( BL \ \mid \ BR ) \ ( TL \quad TR ) }
{ TR \ \mid \ BR \ \mid \ TL \quad BL } \end{align*}

where *TR*, *BR*, *TL*, and *BL* are the top right, bottom right, top left, and bottom left sensors, respectively. COP was tied to targets within the visual display, allowing participants to directly control the position of objects on the screen through changes in their body position and configuration. This link between what the user perceived on the screen and how he or she moved formed the basis of the feedback during both assessment and training. The design also allowed for direct 1:1 mapping of COP displacement as recorded through the WBB and the control of a designated visual object in the game. This real-time biofeedback feedback facilitated an acute awareness of one's own bodily position, an important factor in successful balance training,^14,15^ a facility often lacking in commercially available games.

A range of balance training games and balance assessment tests was designed using this interface, encouraging older adults to explore their limits of stability in a safe environment.

### Balance training system

**FIG. 2.**
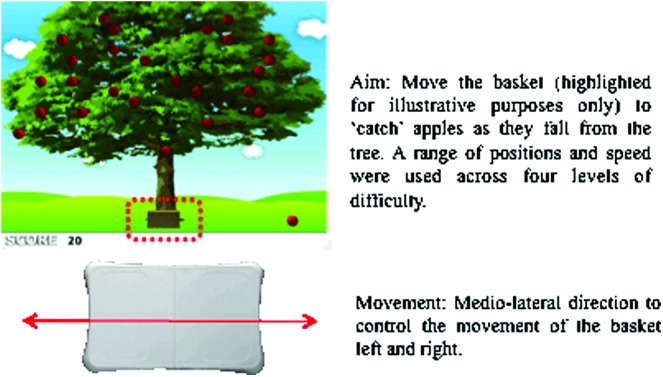
Screenshot and schematic diagrams of the ‘Apple Catch’ game. Participants were required to control the basket using mediolateral movement, to position the basket in beneath the falling apple so they successfully ‘caught’ the apples as they fell from the tree. The speed and location of the apples were systematically varied, leading to increased levels of difficulty. (Color graphics available at www.liebertonline.com/g4h)

**FIG. 3.**
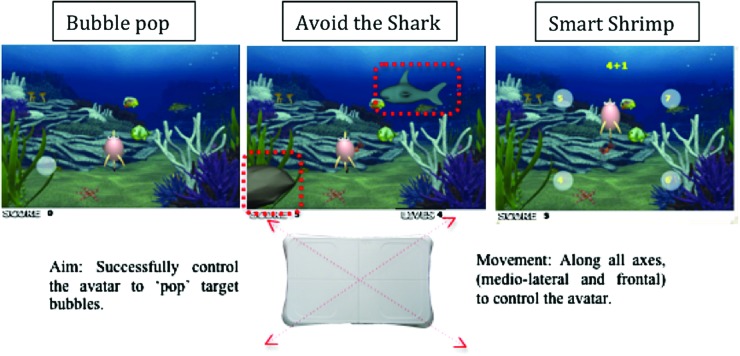
Screenshots and schematic diagrams of ‘Bubble Pop,’ ‘Avoid the Shark,’ and ‘Smart Shrimp.’ These games required participants to successfully ‘pop’ target bubbles by multidimensional control of their center of pressure (both mediolateral and frontal planes). Additional levels of complexity in the ‘Avoid the Shark’ and ‘Smart Shrimp’ games included hiding from a virtual predator and the completing mental tasks. (Color graphics available at www.liebertonline.com/g4h)

For a balance training program to be effective, exercises need to encourage the user to control his or her COP in both static and dynamic settings. Therefore, a range of games was developed, each targeting different components of balance and offering differing levels of complexity. The range of games included ‘Apple Catch,’ ‘Bubble Pop,’ ‘Avoid the Shark,’ and ‘Smart Shrimp,’ each containing four levels of difficulty (Figs. 2 and 3). The first game (‘Apple Catch’; Fig. 2) required participants to control their sway to either the left- or right-hand side and to hold that position to ‘catch’ apples as they fell from a tree. This type of movement strengthened levels of COP displacement within the mediolateral plane and the ability to hold COP at a given position to catch a falling apple. By adjusting the speed, position, and number of apples that dropped from the tree, the postural requirements needed to play the game became progressively more difficult (i.e., apples were positioned more to the extreme lateral positions on the tree). These adaptations to the game mechanics helped challenge the user and invited him or her to push the postural control requirements necessary to succeed in the task.

The remaining games required precise control of COP displacement across both the mediolateral and frontal planes to successfully ‘pop’ static or moving bubbles. Again, game complexity was captured by having the trajectories of these target bubbles becoming progressively more complex, with both bubble size and movement speed varying as a function of level difficulty. This overarching goal was the primary focus of ‘Bubble Pop,’ whereas ‘Avoid the Shark’ and ‘Smart Shrimp’ provided additional levels of movement complexity with additional cognitive demands (Fig. 3). In particular, ‘Avoid the Shark’ required a mix of ballistic controlled shifts in COP to successfully ‘pop’ bubbles and controlled static balance when hiding behind a rock to avoid the virtual predator. It also required the user to switch attention from performing one task (popping bubbles) to another (hiding behind a rock). This integration proved successful, with participants often engaging in challenging behaviors such as crouching or displacing COP to the extreme posterior location to ensure continuation of the game.

In the ‘Smart Shrimp’ game the physical requirements needed to play the game were coupled with cognitive puzzles that incorporated elements of dual-tasking (Fig. 3). The cognitive puzzles included both word completion and mental arithmetic tasks, with participants asked to ‘pop’ the bubble containing the correct answer. In addition, participants were penalized for accidently ‘popping’ a bubble with the wrong answer, enforcing an additional level of inhibitory control. When progressing through the levels of task difficulty, the size and speed of the bubbles varied, the cognitive task increased in difficulty, and the physical challenge required to successfully avoid those bubbles containing the wrong answer became significantly greater. It is important, however, that all games were designed to be sympathetic to the action capabilities of older adults. Indeed, the main game concepts were piloted with a sample of older adults,^43^ and feedback was used to refine the user interface and game parameters.

To add a multisensory dimension to the task, coherent auditory–visual feedback was provided that coincided with movements within the virtual environment. For example, concurrent visual and audio information was provided when ‘apples’ were successfully caught in the ‘Apple Catch’ game, with the sight of each apple disappearing into the basket being accompanied by a thud sound. In the ‘Bubble Pop,’ ‘Avoid the Shark,’ and ‘Smart Shrimp’ games a ‘pop’ sound was simultaneously provided when ‘bubbles’ were successfully hit. Participants were provided with a continuous score throughout the games and were also presented with a final game score at the end of each level. This knowledge of results that relates to balance performance provides a strong motivational component to continually strive to do better in the next game.

### Balance assessment system

A battery of balance assessments provided recordings of COP displacement as a measure of *static* and *dynamic* balance. These assessment tools were designed using commercially available software from the NeuroCom Balance Master as a baseline, with each balance test individually created to match the requirements and outputs generated by this system. The NeuroCom Balance Master is a high-end research-grade balance monitoring system from which measures of postural stability are extracted (e.g., COP trace recording). This system has been shown to have high levels of reliability and validity in a range of populations, including those recovering from brain injury^50^ and stroke,^51^ along with healthy older adults.^52^ The limits of stability test, designed to quantify risk of falls in vulnerable populations, requires individuals to displace their center of mass in a variety of directions. This test has been shown to reliably profile levels of dynamic balance^52,53^ by mapping an individual's limit of stability through measures of maximum excursion, end point excursion, and directional control. These measures were encapsulated in tests of static and dynamic balance and reflected in an overall score of accuracy.

**FIG. 4.**
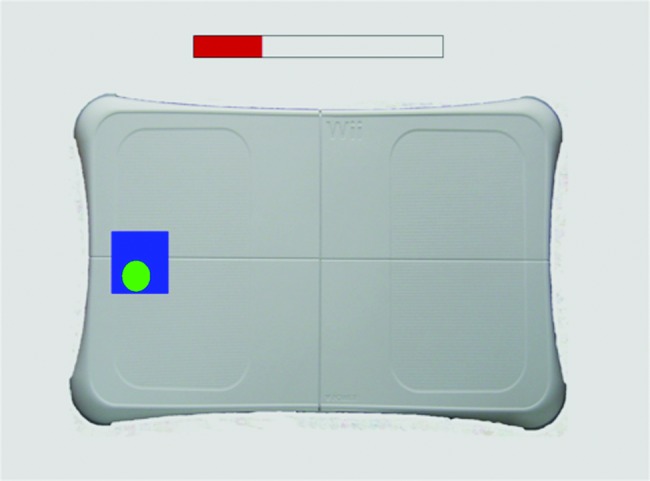
A screenshot from the left static balance test. This required the participant to move his or her weight onto the left leg (left center of pressure displacement). The static balance tests also included targets to the right, bottom, and top of the balance board drawing. (Color graphics available at www.liebertonline.com/g4h)

**FIG. 5.**
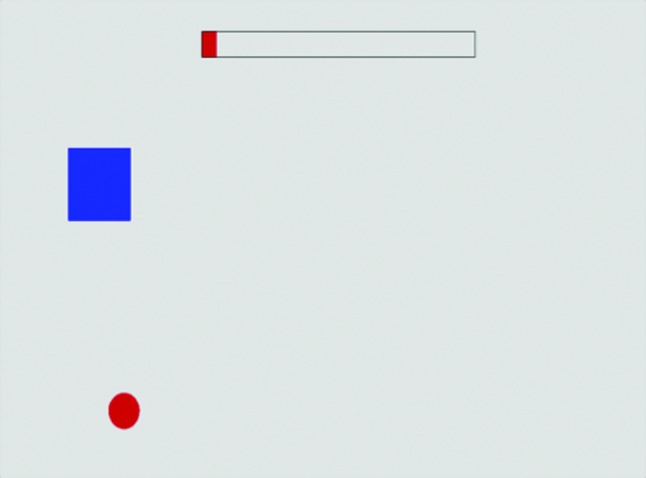
A screenshot of the dynamic balance test. In this test the participant had to hit as many squares as possible in 60 seconds (time dynamically represented by the bar at the top). Once a participant hits a square it disappears, and a new one appears in a different location. (Color graphics available at www.liebertonline.com/g4h)

COP positional information was extracted via the triangulation of data streamed from the four sensor pressures of the WBB using the aforementioned interface. This was then visually represented as a moving circle throughout all levels of balance assessment, providing participants with real-time biofeedback (Figs. 4 and 5). Target locations were displayed (represented as blue squares), which the participant was asked to “aim” for. The COP circle was green when the participant managed to get his or her COP in the target zone and red when the participant was not in the target zone—providing clear feedback on performance and visual guidance (Fig. 4), and a top bar changed dynamically as the test time progressed.

#### Static balance test

The static balance test involved moving the COP (visually represented by a circle) into a specified target zone and keeping it as still as possible for 10 seconds. Depending on the position of the target, this required the participant to shift his or her weight accordingly to ensure his or her COP reached the target position (Fig. 4). The participant was then required to hold his or her COP steady in the target location throughout the trial. At the end of each test, participants were given a score that represented the percentage of time spent in the target area. This was repeated three times for each target location (center, anterior, right, posterior, and left—presented in this sequence) before the average score was automatically calculated and stored in a central SQLite database, along with kinematic data. A standard C programming tool, the SQLite database allowed data to be easily stored, concatenated, and retrieved when required.

#### Dynamic balance test

The dynamic balance test required participants to rapidly, and accurately, displace their COP to target locations. In particular, this test involved trying to ‘hit’ as many target locations as possible within a 60-second time frame (Fig. 5). The position of the specified target zone was systematically varied between posterior (center) to anterior (anterior left, center, and right) locations to allow assessment of dynamic balance during transition between anterior and posterior balance. Participants could not progress until they hit the square that was displayed. At the end of 60 seconds, the participants were given a score representing the total number of squares hit.

#### Test scoring

At the end of each test, participants received a score that represented the percentage of time spent in the target area, or the number of targets successfully ‘hit.’ Scores therefore represented levels of COP spatial accuracy and directly reflected levels of postural control and stability. All scores were stored for further analysis in a central SQlite database. Following completion, raw data were extracted for analysis; in this instance, data were converted to percentage change between Session 1 and Session 2.

### Procedure and measures

**FIG. 6.**
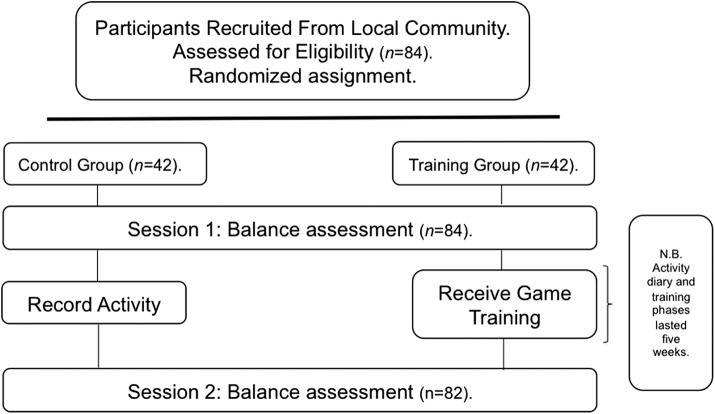
Schematic outline of the procedure used. As outlined, 84 participants were initially recruited from the local community before being screened for inclusion criteria and then being randomly assigned to either a control (*n* = 42) or training (*n* = 42) group. All participants then completed Session 1, during which time various balance and balance confidence measures were taken. The control group then completed an activity diary for a period of 5 weeks, whereas the training group received balance game training—10 sessions over the same 5-week period. Upon completion, all participants in Session 2 received the same range of balance assessment measures used in Session 1. Note that two participants failed to successfully complete the game training because of illness.

All participants were screened for inclusion criteria (see Participants section), including cognitive impairment using the MMSE,^49^ before being randomly assigned to either a control or experimental group. Balance confidence was measured using the Activities-Specific Balance Confidence Scale,^54^ followed by levels of functional balance, which were taken using static and dynamic balance tests described above and the BBS^40^ (Session 1). Following completion of Session 1 assessment, the experimental group took part in a structured balance training program using the tailored balance training games (as outlined above). All experimental participants completed a total of 10 training sessions, lasting a minimum of 30 minutes per session, over a course of 5 weeks. During this time, the control group logged daily activity. Following completion of the course, levels of balance confidence and functional balance were reassessed in all participants (both control and experimental groups) (Session 2). Figure 6 provides a schematic representation of this structure.

Following completion of the study, both the control and experimental groups were further subdivided into those individuals at low and high risk of falls according to user guidelines provided for the BBS by Berg et al.^40^: a score of ≤45 on the BBS was indicative of high risk of falls. This further classification facilitated an understanding of the usability of such a system with all levels of functional ability, as well as the impact of such balance training programs for those individuals at most risk.

All control participants recorded levels of daily activity using a record diary. This paper-based diary included predefined terms to guide completion, asking participants to record Light, Medium, and Heavy activities, along with the data and duration of the activity. These categories were designed using the “Wii Fit” diary, with clear examples of each provided for guidance. Examples of light activities included cooking, laundry, and light stretches, medium activities included gardening, cleaning, and childcare, and heavy activities included bowling, dancing, and swimming. The experimental group took part in structured training twice a week. The primary researcher remained with each participant, tailoring the training session to the individual's needs and functional ability, while providing feedback on previous scores and activity for reference. Participants undertaking game training abstained from additional structured exercises.

### Data extraction and analysis

All standardized tests, such as the BBS,^40^ were scored according to the author guidelines. Where appropriate, initial levels of functional balance (BBS during Session 1) were controlled for by using analysis of covariance. Balance was also measured through recordings of COP displacement via the aforementioned balance tests. Participant scores in *static* balance tests were averaged across three trials for targets in center, anterior, right, posterior, and left locations (each test location was presented in this sequential order for the static test). Scores for all balance tests (custom-made and BBS) were further converted into a percentage change in score between Sessions 1 and 2 using the following equation:

\begin{align*} & ([ \rm {Score \ at \ Time} \ 2 - { \rm Score \ at
\ Time} \ 1 ] / \\ & {  \rm Maximum \ Test \ Score} ) \times 100 =
{ \rm Percentage \ Change} \end{align*}

An alpha threshold of 0.5 was applied throughout.

## Results

### Control information

Both the control and experimental groups were adequately matched on pre-Berg scores (*P* = 0.768), MMSE (*P* = 0.895), and age (*P* = 0.956) (Table 1).

**Table 1.** Summary Characteristics of the Overall Experimental and Control Groups and the Subgroups of Those Individuals Deemed at High and Low Risk of Falling

			*Berg*		*ABC*	
*Group type*	*Age (years)*	*MMSE*	*Pre*	*Post*	*Pre*	*Post*
Experimental	77.18 (6.59)	28.20 (1.32)	45.93 (6.84)	50.1 (6.40)^c^	65.25 (20.5)	75.25 (15.07)^c^
Control	76.62 (7.28)	28.06 (1.56)	46.45 (9.08)	46.90 (9.21)	61.20 (28.42)	67.92 (21.49)
High risk						
Experimental	77.73 (8.01)	28.00 (1.25)	38.87 (5.80)	44.73 (7.31)^a^	45.89 (13.04)	63.60 (10.51)^c^
Control	79.00 (7.03)	27.50 (1.31)	35.42 (10.26)	36.67 (10.64)	40.00 (22.00)	50.58 (16.65)^a^
Low risk						
Experimental	76.83 (5.64)	28.32 (1.38)	50.16 (2.54)	53.32 (2.54)^a^	76.87 (14.51)	82.25 (12.99)^a^
Control	75.67 (7.27)	28.24 (1.61)	50.87 (2.45)	51.00 (4.03)	69.67 (26.42)	74.86 (19.33)

Data are mean (standard deviation) values. Levels of cognitive ability were initially assessed using the Mini Mental State Examination (MMSE). Levels of functional balance were measured using a standardized clinical tool (the Berg Balance Scale). Balance confidence was assessed using the Activities-specific Balance Confidence Scale (ABC). Scores for functional balance and balance confidence prior to and following completion of the experimental or control phase are provided.

^a^ *P* < 0.05, ^b^*P* < 0.005, ^c^*P* < 0.001.

### Balance confidence

As shown in Table 1, both the control and experimental groups reported similar levels of balance confidence during Session 1. It is interesting that both groups displayed a trend for increased levels of confidence following the intervention or control phase. However, this increase was marked for the experimental group. An analysis of covariance, controlling for levels of functional balance as measured using the BBS during Session 1, found a significant main effect of Session (*F*_1, 79_ = 14.013, *P* < 0.001, η^2^ = 0.151), whereas the main effect of Group approached significance (*F*_1, 79_ = 3.640, *P* = 0.06, η^2^ = 0.044). *Post hoc* analysis (paired *t* tests) revealed a significant effect of session on Activities-Specific Balance Confidence Scale scores for the experimental group (*P* < 0.001) but not the control group (*P* = 0.021), implying an effect of balance game training. Note that a reduction in the alpha threshold, to 0.01, was applied due to a violation of Levene's test of equal variances.^55^

### Functional balance: BBS

As demonstrated in Table 1, both the experimental and control groups displayed similar levels of functional balance during Session 1. However, the experimental group displayed a significant increase following balance training (*P* < 0.001). Despite showing a trend for increased functional balance, the control group failed to mirror this result (*P* = 0.518).

### Postural control: custom-made balance tests

**FIG. 7.**
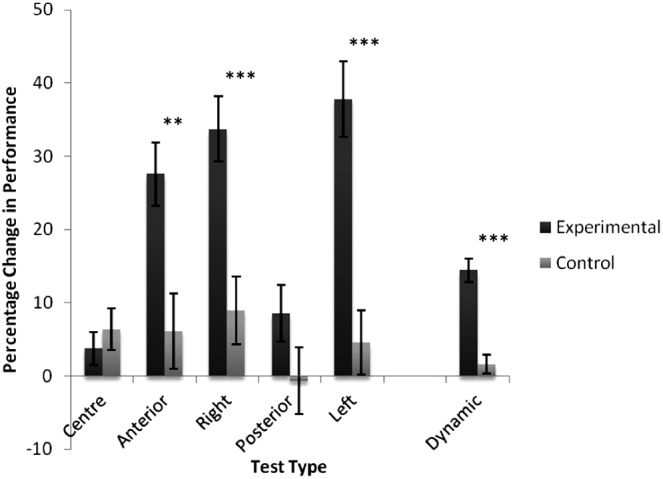
Summary of the percentage change in balance scores calculated for both groups between Sessions 1 and 2. Error bars represent standard error of the mean. Note that scores for the percentage change in the center position were low because most participants were close to 100 percent in Session 1. **P < 0.005, ***P < 0.001.

Both groups demonstrated an overall trend for increased performance in levels of COP displacement (Fig. 7 and Table 2). However, this increase was pronounced for the experimental group. A significant main effect of Group was found through multivariate analysis of variance (Wilks' lambda = 0.709, *F*_5, 76_ = 6.225, *P* < 0.001, η^2^ = 0.291), implying an underlying positive effect of balance game training on postural control. This effect was observed on the following COP test locations: anterior (*P* = 0.002), right (*P* < 0.001), and left (*P* < 0.001). The effect failed to reach significance for both center (*P* = 0.468) and posterior (*P* = 0.124) tests. The experimental group also showed a significant increase in levels of performance on the dynamic test relative to controls (*F*_1, 80_ = 39.54, *P* < 0.001, η^2^ = 0.331), again implying a significant impact of balance training on levels of dynamic postural control.

**Table 2.** Summary Raw Balance Data for Kinematic Static Balance Tests (i.e., Score Representing Average Time Spent in Target Location)

	*Center*		*Anterior*		*Right*		*Posterior*		*Left*	
*Group type*	*Pre*	*Post*	*Pre*	*Post*	*Pre*	*Post*	*Pre*	*Post*	*Pre*	*Post*
Experimental	88.99 (13.31)	92.75 (8.25)	50.28 (26.94)	77.86 (15.68)^a^	17.48 (22.91)	51.19 (28.97)^b^	74.07 (24.18)	82.67 (16.62)	21.63 (29.50)	59.38 (29.71)^b^
Control	82.19 (18.59)	88.60 (12.38)	60.02 (28.17)	66.12 (26.61)	27.14 (27.44)	36.07 (32.47)	81.24 (23.43)	80.60 (22.81)	24.23 (30.08)	28.85 (31.74)
High risk										
Experimental	89.07 (17.21)	89.01 (9.65)	43.07 (31.96)	82.82 (11.66)	20.60 (23.77)	57.45 (29.80)^a^	73.13 (30.58)	83.78 (17.58)	18.87 (29.74)	63.18 (29.59)^a^
Control	81.25 (20.32)	88.33 (9.17)	59.50 (23.09)	67.75 (24.26)	27.17 (31.83)	37.92 (34.38)	69.75 (28.29)	69.75 (30.83)	22.42 (30.22)	26.58 (33.01)
Low risk										
Experimental	88.94 (10.74)	94.99 (6.50)	54.61 (23.06)	74.86 (17.18)	15.61 (22.67)	47.44 (28.39)^a^	74.63 (20.10)	82.00 (16.35)	23.29 (29.83)	57.10 (30.16)^a^
Control	82.57 (18.20)	88.71 (13.59)	60.22 (30.32)	65.47 (27.86)	27.14 (26.08)	35.34 (32.25)	85.84 (19.90)	84.94 (17.51)	24.95 (30.50)	29.75 (31.76)

Data are mean (standard deviation) values. Overall summary data are displayed for both the experimental and control groups. Results are further deconstructed to display overall performance in individuals deemed at high and low risk of falling. As outlined, the battery of static balance tests included a range of test locations: center, anterior, right, posterior, and left. Summary scores are provided for each test location, prior to and following the experimental or control session.

^a^ *P* < 0.005, ^b^*P* < 0.001.

### Subgroup analysis—impact on high-risk participants

Participants were further divided into a subgroup categorized as at high risk of falls according to user guidelines provided for the BBS by Berg et al.^40^ (a score of ≤45 on the BBS). This produced a subexperimental high-risk group of 15 participants (2 males, 13 females; 77.73 ± 8.01 years old) and a subcontrol high-risk group of 12 participants (6 males, 6 females; 79.00 ± 7.03 years old) (Table 1).

**FIG. 8.**
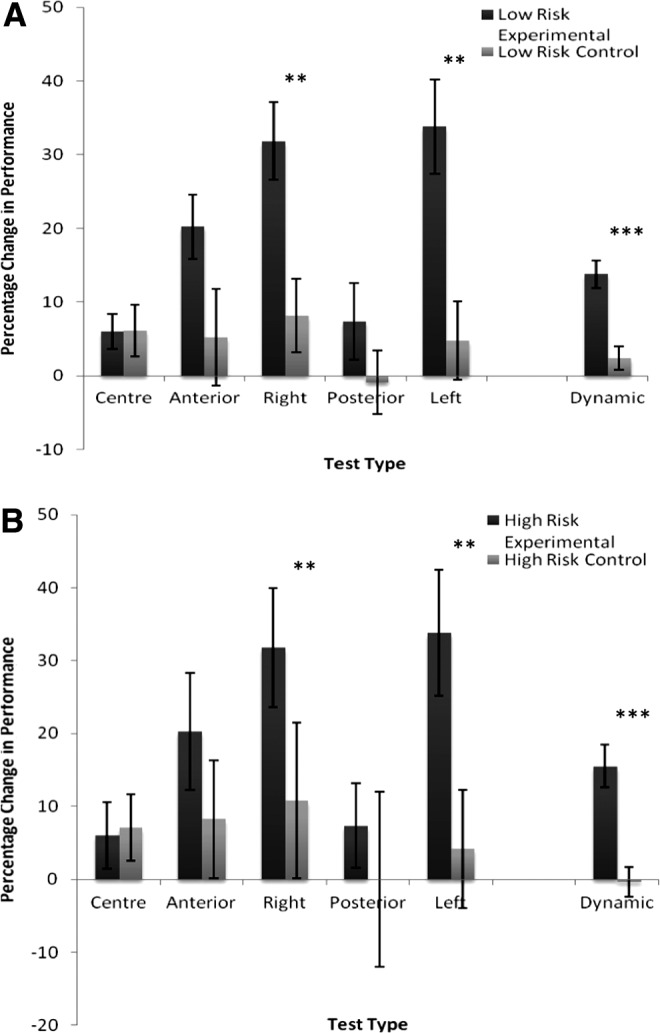
A summary of the characteristics of the changes in balance performance (static and dynamic) observed in the experimental and control participants, who were classed as at **(A)** a high and **(B)** a low risk of falls. The experimental group displayed marked increases in levels of balance performance following game training. Despite a general trend for increased balance ability within the control group, this failed to reach significance. **P < 0.005, ***P < 0.001.

Balance game training resulted in a pronounced increase in levels of functional balance as measured through the BBS (*F*_1, 25_ = 4.270, *P* = 0.049, η^2^ = 0.146), custom-made static (Wilks' lambda = 0.449, *F*_5, 20_ = 4.903, *P* = 0.004, η^2^ = 0.551), and dynamic (*F*_1, 25_ = 18.04, *P* < 0.001, η^2^ = 0.419) balance tests for this subgroup of high-risk individuals. Mirroring the overall results, increases were prominent in performance on the anterior (*P* = 0.086), right (*P* = 0.002), and left (*P* = 0.001) test locations (Fig. 8).

Similar analyses indicated significant gains within the experimental low-risk group (3 males, 22 females; 76.83 ± 5.64 years old) in relation to their respective control group (14 males, 16 females; 75.67 ± 7.28 years old). However, no significant difference was observed between gains made by the two experimental groups (i.e., low-risk versus high-risk), implying suitability of these balance games for individuals at all levels of ability.

It is interesting that all high-risk individuals demonstrated a significant increase in levels of balance confidence following completion of the study (experimental, *P* < 0.001; control, *P* = 0.029). In contrast, only the low-risk experimental group demonstrated a significant increase in balance confidence (*P* = 0.007; control, *P* = 0.156).

## Discussion

Epitomizing the notion of enjoyment, fun, and competitiveness, gameplay can provide an immersive platform to motivate and engage individuals of all ages. This study demonstrates the potential of gaming technology to provide an immersive, effective balance training tool for the older population by designing games that encourage users to control COP (balance) in a range of tasks.

The positive effect of structured balance game training was demonstrated across levels of both functional balance and balance confidence, which was consistent for groups at both high and low risk of falls. This is of particular importance. Although similar studies demonstrated the *potential* of commercial Nintendo games to improve physical function in older adults,^23–32^ results are limited and inconsistent in high-risk groups.^24,38^ High-risk individuals demonstrated an average increase of 5.87 points on the BBS (Table 1), with an improvement of 4 points on average for a similar study using commercially available games.^24^ Note that the minimal detectable change in a BBS score is 5 points.^39,41^ Moreover, Agmon et al.^24^ used a more liberal threshold for high-risk classification (i.e., setting a BBS threshold of ≤52), which may have artificially inflated the ability of the high-risk group. Indeed, when a similar conservative BBS threshold for high-risk categorization is used with commercially available games, no significant increase in BBS scores was observed for high-risk adults.^38^ This pattern could be attributed to the use of *commercially available* games, reinforcing the need for balance training games that are tailored to the needs of older adults. Our games were designed to be sympathetic to the movement capabilities of older adults, and thus inclusive, while maintaining a direct one-to-one mapping between the movement of a visual object presented in the game and actual COP displacement. It is worth highlighting that these important features are often lacking in commercially available games.^56^ Overall, the results demonstrate both an increase in functional balance and a simultaneous reduction in fall risk (an approximately 6% reduction in fall risk per 1-point increase^57^).

Improvements across both static and dynamic balance tests demonstrate the versatility of the system. In particular, improvements in dynamic balance imply a significant increase in participants' limits of stability, whereas the pattern of static performance suggests that the balance games are targeting specific movement capabilities in older adults. Moreover, those that appear to be targeted, such as mediolateral displacement, have been connected to fall risk.^58^ In contrast, participants displayed limited improvement during the static center and posterior tests between Sessions 1 and 2. This could be an artifact inherent to the scoring method used, which may not be sensitive enough to changes in these testing locations. COP sway velocity and/or trace could be used in future studies to explore this further.

The multifaceted nature of improvement demonstrated by the experimental group further supports the potential of gaming as a platform for balance training. Psychological improvements in self-reported balance confidence were supported by anecdotal reports of improved mood, self-confidence, and self-worth. It is interesting that both the experimental and control groups displayed a notable improvement in levels of reported balance confidence. In particular, results indicate that those deemed high risk showed significant increases in levels of balance confidence following completion of either the intervention or control phase. As mentioned, the control phase required participants to record levels of physical activity. This raises the question, are we naturally inflating levels of perceived confidence by simply raising awareness of physical activity? This is problematic given the predominance of educational schemes such as the “Stay on Your Feet” program of Western Australia,^59^ which use similar techniques to improve levels of balance confidence. Without parallel increases in levels of functional balance, this increase in levels of balance confidence may be cause for concern.

Currently, there is no comprehensive method to diagnose and prevent falls in older adults, and existing intervention strategies prevent less than 30 percent of falls.^60^ Although recent studies have explored the potential of using high-end research-grade balance technology to profile and train functional balance control, this technology is often unattainable because of financial and practical limitations. By designing ‘serious games’ that are simultaneously sensitive to older adults' movement capabilities, we have delivered an engaging, effective exercise-based balance therapy. Moreover, coupling commercially available hardware with custom-made software programs, we have designed a portable balance training and assessment tool—a vital step toward the large-scale, effective implementation of such systems.

## References

[1] Winter DA, Patla AE, Frank JS Assessment of balance control in humans. Med Prog Technol 1990; 16:31–51 2138696

[2] Mahoney JR, Holtzer R, Verghese J Visual-somatosensory integration and balance: Evidence for psychophysical integrative differences in aging. Multisens Res 2014; 27:17–42 25102664 10.1163/22134808-00002444 PMC4280078

[3] Whipple R, Wolfson L, Derby C, et al. Altered sensory function and balance in older persons. J Gerontol 1993; 48(Spec No):71–76 8409244 10.1093/geronj/48.special_issue.71

[4] Masani K, Vette AH, Kouzaki M, et al. Larger centre of pressure minus centre of gravity in the elderly induces larger body acceleration during quiet standing. Neurosci Lett 2007; 422:202–206 17611029 10.1016/j.neulet.2007.06.019

[5] Abrahamova D, Hlavacka F Age-related changes of human balance during quiet stance. Physiol Res 2008; 57:957–964 18052683 10.33549/physiolres.931238

[6] King MB, Judge JO, Wolfson L Functional base of support decreases with age. J Geronotol 1994; 49:M248–M263 10.1093/geronj/49.6.m258 7963278

[7] Newton R Validity of the multi-directional reach test: A practical measure for limits of stability in older adults. J Gerontol A Biol Sci Med Sci 2001; 56:M248–M252 11283199 10.1093/gerona/56.4.m248

[8] Hadjistavropoulos T, Delbaere K, Fitzgerald TD Reconceptualizing the role of fear of falling and balance confidence in fall risk. J Aging Health 2011; 23:3–23 20852012 10.1177/0898264310378039

[9] Tromp AM, Pluijm SMF, Smit JH, et al. Fall-risk screening test: A prospective study on predictors for falls in community-dwelling elderly. J Clin Epidemiol 2001; 54:837–844 11470394 10.1016/s0895-4356(01)00349-3

[10] NICE Guidelines [CG161]. Falls: The Assessment and Prevention of Falls in Older People. London: National Institute for Clinical Excellence; 2013 25506960

[11] National Center for Injury Prevention and Control, Centers for Disease Control and Prevention. Web-Based Injury Statistics Query and Reporting System (WISQARS). 2013 www.cdc.gov/injury/wisqars/ (accessed 731, 2014)

[12] NICE Guidelines [CG21]. Falls: The Assessment and Prevention of Falls in Older People. London: National Institute for Clinical Excellence; 2004

[13] Rose DJ Preventing falls among older adults: No “one size suits all” intervention strategy. J Rehabil Res Dev 2008; 45:1153–1166 19235117

[14] Horak FB, Shupert CL, Mirka A Components of postural dyscontrol in the elderly: A review. Neurobiol Aging 1989; 10:727–738 2697808 10.1016/0197-4580(89)90010-9

[15] Pai YC, Wening JD, Runtz EF, et al. Role of feedforward control of movement stability in reducing slip-related balance loss and falls among older adults. J Neurophysiol 2003; 90:755–762 12904492 10.1152/jn.01118.2002

[16] Maclean N, Pound P A critical review of the concept of patient motivation in the literature on physical rehabilitation. Soc Sci Med 2000; 50:495–506 10641802 10.1016/s0277-9536(99)00334-2

[17] Nilson NC, Stefania S, Rolf N Gameplay as a source of intrinsic motivation for individuals in need of ankle training or rehabilitation. Presence 2012; 21:69–84

[18] Bryanton C, Bossé J, Brien M, et al. Feasibility, motivation, and selective motor control: Virtual reality compared to conventional home exercise in children with cerebral palsy. Cyberpsychol Behav 2006; 9:123–128 16640463 10.1089/cpb.2006.9.123

[19] Betker AL, Desai A, Nett C, et al. Game-based exercises for dynamic short sitting balance rehabilitation of people with chronic spinal cord and traumatic brain injuries. Phys Ther 2007; 87:1389–1398 17712036 10.2522/ptj.20060229

[20] Sihvonen SE, Sipilä S, Era PA Changes in postural balance in frail elderly women during a 4-week visual feedback training: A randomized controlled trial. Gerontology 2004; 50:87–95 14963375 10.1159/000075559

[21] Wolf SL, Barnhart HX, Ellison GL, Coogler CE The effect of Tai Chi Quan and computerized balance training on postural stability in older subjects. Atlanta FICSIT Group. Frailty and Injuries: Cooperative Studies on Intervention Techniques. Phys Ther 1997; 77:371–381 9105340 10.1093/ptj/77.4.371

[22] Lajoie Y Effect of computerized feedback postural training on posture and attentional demands in older adults. Aging Clin Exp Res 2004; 16:363–368 15636461 10.1007/BF03324565

[23] Pigford T, Andrews AW Feasibility and benefit of using the Nintendo Wii Fit for balance rehabilitation in an elderly patient experiencing recurrent falls. J Student Phys Ther Res 2010; 2:12–20

[24] Agmon M, Perry CK, Phelan E, et al. A pilot study of Wii Fit Exergames to improve balance in older adults. J Geriatr Phys Ther 2011; 34:161–167 22124415 10.1519/JPT.0b013e3182191d98

[25] Williams B, Doherty NL, Bender A, et al. The effect of Nintendo Wii on balance: A pilot study supporting the use of the Wii in occupational therapy for the well elderly. Occup Ther Health Care 2011; 25:131–139 23899030 10.3109/07380577.2011.560627

[26] Bateni H Changes in balance in older adults based on use of physical therapy vs the Wii Fit gaming system: A preliminary study. Physiotherapy 2012; 98:211–216 22898577 10.1016/j.physio.2011.02.004

[27] Franco JR, Jacobs K, Inzerillo C, Kluzik J The effect of the Nintendo Wii Fit and exercise in improving balance and quality of life in community dwelling elders. Technol Health Care 2012; 20:95–115 22508022 10.3233/THC-2011-0661

[28] Padala KP, Padala PR, Malloy TR, et al. Wii-Fit for improving gait and balance in an assisted living facility: A pilot study. J Aging Res 2012; 2012:597573 22745909 10.1155/2012/597573 PMC3382377

[29] Rendon AA, Lohman EB, Thrope D, et al. The effect of virtual reality gaming on dynamic balance in older adults. Age Ageing 2012; 41:549–552 22672915 10.1093/ageing/afs053

[30] Toulotte C, Toursel C, Olivier N Wii Fit^®^ training vs. adapted physical activities: Which one is the most appropriate to improve the balance of independent senior subjects? A randomized controlled study. Clin Rehabil 2012; 26:827–835 22324055 10.1177/0269215511434996

[31] Goble DJ, Cone BL, Fling BW Using the Wii Fit as a tool for balance assessment and neurorehabilitation: The first half decade of “Wii-search.” J Neuroengin Rehabil 2014; 11:12 10.1186/1743-0003-11-12 PMC3922272 24507245

[32] Nicholson VP, McKean M, Lowe J, et al. Six weeks of unsupervised Nintendo Wii Fit gaming is effective at improving balance in independent older adults. J Aging Phys Act 2015; 23:153–158 24589631 10.1123/japa.2013-0148

[33] Saposnik G, Levin M Virtual reality in stroke rehabilitation: A meta-analysis and implications for clinicians. Stroke 2011; 42:1380–1386 21474804 10.1161/STROKEAHA.110.605451

[34] Levac DE, Miller P, Missiuna C Usual and virtual reality video game-based physiotherapy interventions for children and youth with acquired brain injuries. Phys Occup Ther Pediatr 2012; 32:180–195 21942894 10.3109/01942638.2011.616266

[35] Levac DE, Miller PA Integrating virtual reality video games into practice: Clinicans' experiences. Physiother Theory Pract 2013; 29:504–512 23362843 10.3109/09593985.2012.762078

[36] Ambrose AF, Paul G, Hausdoff JM Risk factors for falls among older adults: A review of the literature. Maturitas 2013; 75:51–61 23523272 10.1016/j.maturitas.2013.02.009

[37] Laver K, George S, Thomas S, et al. Virtual reality for stroke rehabilitation. Eur J Phys Rehabil Med 2012; 48:523–530 22713539

[38] Bainbridge S, Keeley B, Oriel K The effects of the Nintendo Wii Fit on community-dwelling older adults with perceived balance deficits: A pilot study. Phys Occup Ther Geriatr 2011; 29:126–135

[39] Berg KO, Wood-Dauphine S, Williams JI The Balance Scale: Reliability assessment with elderly residents and patients with an acute stroke. Scand J Rehabil Med 1995; 27:27–36 7792547

[40] Berg KO, Wood-Dauphinee SL, Williams JI, Maki B Measuring balance in the elderly: Validation of an instrument. Can J Public Health 1992; 83(Suppl 2) S7–S11 1468055

[41] Stevenson TJ Detecting change in patients with stroke using the Berg Balance Scale. Aust J Physiother 2001; 47:29–38 11552860 10.1016/s0004-9514(14)60296-8

[42] Clark RA, Bryant AL, Pua Y, et al. Validity and reliability of the Nintendo Wii Balance Board for assessment of standing balance. Gait Posture 2010, 31:307–310 20005112 10.1016/j.gaitpost.2009.11.012

[43] Young W, Ferguson S, Brault S, Craig C Assessing and training standing balance in older adults: A novel approach using the ‘Nintendo Wii’ Balance Board. Gait Posture 2011; 33:303–305 21087865 10.1016/j.gaitpost.2010.10.089

[44] Podsiadlo D, Richardson S The timed “Up & Go”: A test of basic functional mobility for frail elderly persons. J Am Geriatr Soc 1991; 39:142–148 1991946 10.1111/j.1532-5415.1991.tb01616.x

[45] Brauer S, Burns Y, Galley P Lateral reach: A clinical measure of medio-lateral postural stability. Physiother Res Int 1999; 4:81–88 10444759 10.1002/pri.155

[46] Newton R Validity of the multi-directional reach test: A practical measure for limits of stability in older adults. J Gerontol 2001; 56A:M248–M252 10.1093/gerona/56.4.m248 11283199

[47] Neurocom International Inc. NeuroCom Balance Master. www.natus.com/index.cfm?page=products_1&crid=271 (accessed 621, 2015)

[48] Reed-Jones RJ, Dorgo S, Hitchings MK, Bader JO WiiFit Plus balance test scores for the assessment of balance and mobility in older adults. Gait Posture 2012; 36:430–433 22534562 10.1016/j.gaitpost.2012.03.027 PMC3407275

[49] Folstein MF, Folstein SE, McHugh PR “Mini-mental state.” A practical method for grading the cognitive state of patients for the clinician. J Psychiatr Res 1975; 12:189–198 1202204 10.1016/0022-3956(75)90026-6

[50] Newstead AH, Hinman MR, Tomberlin JA Reliability of the Berg Balance Scale and Balance Master limits of stability tests for individuals with brain injury. J Neurol Phys Ther 2005; 29:18–23 16386157 10.1097/01.npt.0000282258.74325.cf

[51] Liston RAL, Brouwer BJ Reliability and validity of measures obtained from stroke patients using the Balance Master. Arch Phys Med Rehabil 1996; 77:425–430 8629916 10.1016/s0003-9993(96)90028-3

[52] Clark S, Rose DJ, Fujimoto K Generalizability of the limits of stability test in the evaluation of dynamic balance among older adults. Arch Phys Med Rehabil 1997; 78:1078–1084 9339156 10.1016/s0003-9993(97)90131-3

[53] Clark S, Rose DJ Evaluation of dynamic balance among community-dwelling older adult fallers: A generalizability study of the limits of stability test. Arch Phys Med Rehabil 2001; 82:468–474 11295006 10.1053/apmr.2001.21859

[54] Powell LE, Myers AM The Activities-specific Balance Confidence (ABC) Scale. J Gerontol A Biol Sci Med Sci 1995; 50A:M28–M34 7814786 10.1093/gerona/50a.1.m28

[55] Tabachnick BG, Fidell LS Using Multivariate Statistics. New York: HarperCollins College Publishers; 1996

[56] Awad M, Ferguson S, Craig C Designing games for older adults: An affordance based approach. In: 2014 IEEE 3rd International Conference Serious Games and Applications for Health (SeGAH) New York: IEEE; 2014:1–7

[57] Shumway-Cook A, Baldwin M, Polissar NL, Gruber W Predicting the probability for falls in community-dwelling older adults. Phys Ther 1997; 77:812–819 9256869 10.1093/ptj/77.8.812

[58] Maki BE Gait changes in older adults: Predictors of falls or indicators of fear. J Am Geriatr Soc 1997; 45:313–320 9063277 10.1111/j.1532-5415.1997.tb00946.x

[59] Kempton A, van Beurden E, Sladden T, et al. Older people can stay on their feet: Final results of a community-based falls prevention programme. Health Promot Int 2000; 15:27–33

[60] Parry SW, Frearson R, Steen N, et al. Evidence-based algorithms and the management of falls and syncope presenting to acute medical services. Clin Med 2008; 8:157–162 18478859 10.7861/clinmedicine.8-2-157 PMC4953000

